# Supplementary material for: Label-Free Evaluation of Lung and Heart Transplant Biopsies Using Tissue Autofluorescence-Based Virtual Staining
Source: BME Front. 2025 Jul 2;6:0151. doi: 10.34133/bmef.0151 (PMC12217214; doi:10.34133/bmef.0151)
Supplement: Supplementary 1 — Figs. S1 to S23 [file bmef.0151.f1.pdf]

Supplementary Information for

Label-free evaluation of lung and heart transplant biopsies using tissue autofluorescence-based virtual staining

Yuzhu Li[†,1,2,3], Nir Pillar[†,1,2,3], Tairan Liu[1,2,3], Guangdong Ma[1], Yuxuan Qi[4], Kevin de Haan[1,2,3], Yijie Zhang[1,2,3], Xilin Yang[1,2,3], Adrian J. Correa[5], Guangqian Xiao[5], Kuang-Yu Jen[6], Kenneth A. Iczkowski[6], Yulun Wu[7], William Dean Wallace[5], and Aydogan Ozcan[*,1,2,3,8]

[1]Electrical and Computer Engineering Department, University of California, Los Angeles, CA, 90095, USA.
[2]Bioengineering Department, University of California, Los Angeles, CA, 90095, USA.
[3]California NanoSystems Institute (CNSI), University of California, Los Angeles, CA, 90095, USA.
[4]Department of Computer Science, University of California, Los Angeles, 90095, USA.
[5]Department of Pathology, Keck School of Medicine, University of Southern California, Los Angeles, CA, 90033, USA.
[6]Department of Pathology and Laboratory Medicine, School of Medicine, University of California, Davis, Sacramento, CA, 95817, USA.
[7]Department of Mathematics, University of California, Los Angeles, CA, 90095, USA.
[8]Department of Surgery, University of California, Los Angeles, CA, 90095, USA.

*Correspondence: Aydogan Ozcan, ozcan@ucla.edu
[†] Equal contributing authors

Supplementary Figures

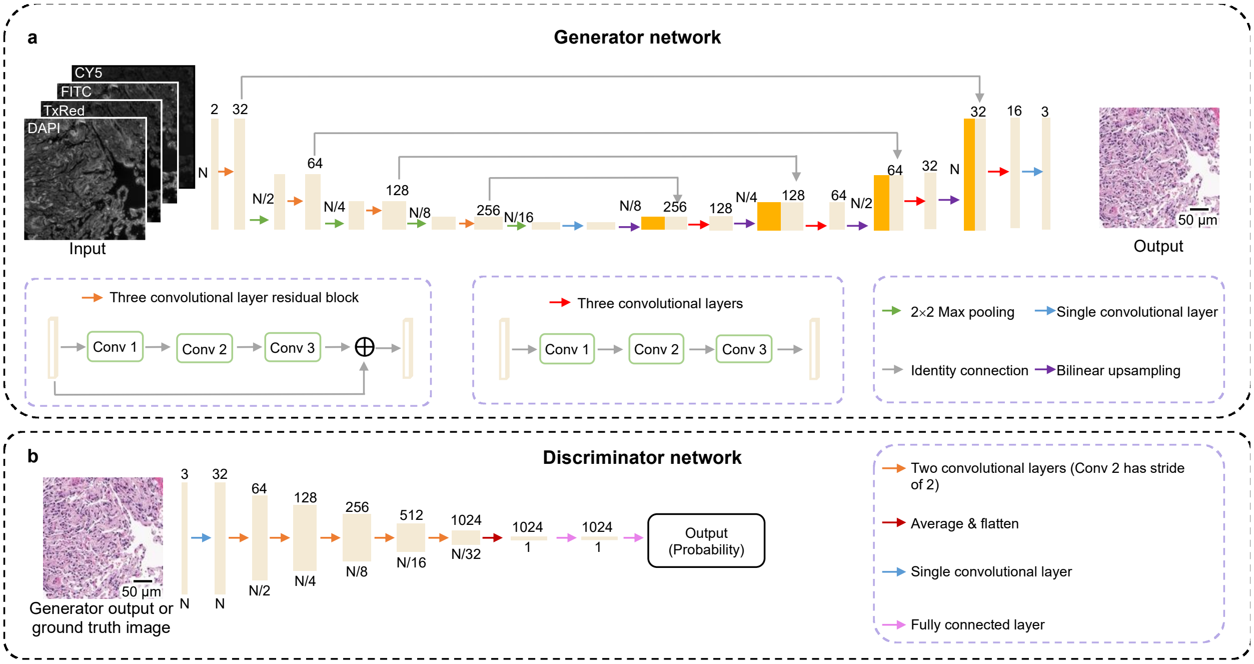

Supplementary Figure 1. The structurally-conditioned GAN framework used for training the virtual staining DNN models for lung and heart transplant biopsies. (a) The generator uses a U-net structure to map the label-free autofluorescence images into bright-field equivalent H&E/MT/EVG images. (b) The discriminator is employed to differentiate between virtually stained images produced by the generator model and the actual histochemically stained ground truth images.

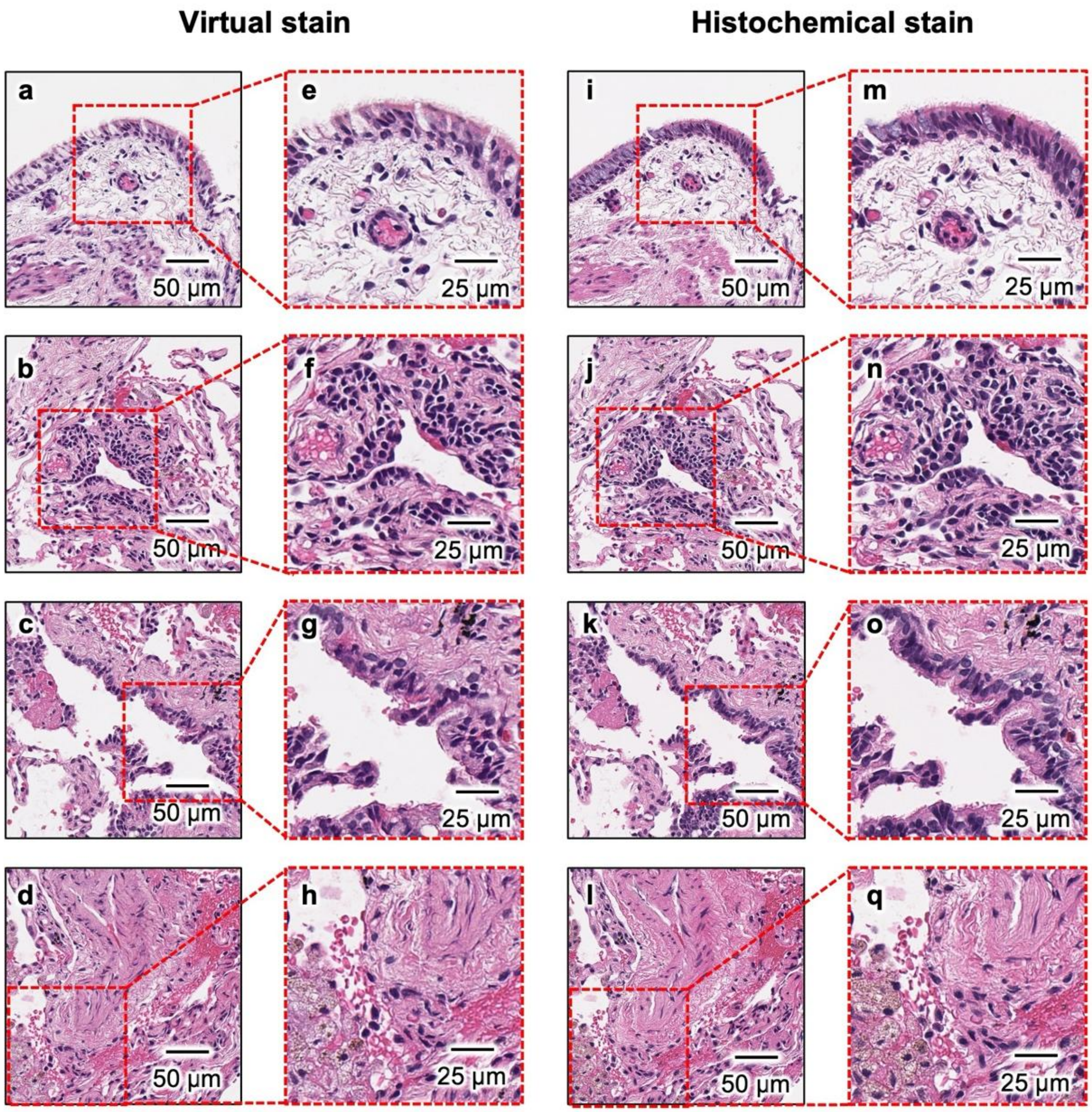

Supplementary Figure 2. Expanded views of the zoomed-in H&E-stained regions from main text Fig. 2. (a–d) Virtually stained H&E regions corresponding to Fig. 2(c1–f1) in the main text. (e–h) Higher-magnification insets of the areas outlined in (a–d). (i–l) Histochemically stained H&E regions corresponding to Fig. 2(c2–f2) in the main text. (m–q) Higher-magnification insets of the areas outlined in (i–l).

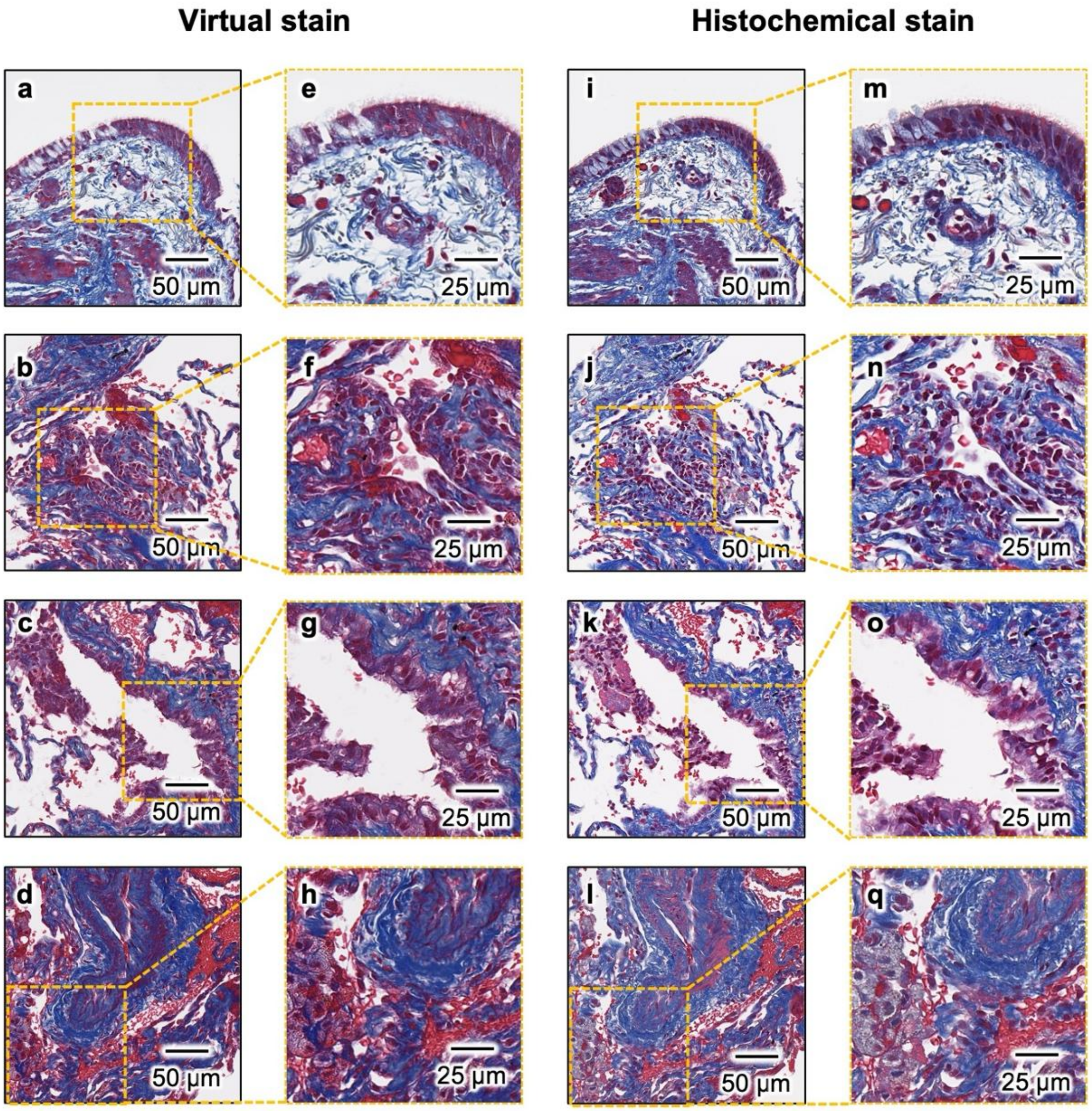

Supplementary Figure 3. Expanded views of the zoomed-in MT-stained regions from main text Fig. 2. (a–d) Virtually stained MT regions corresponding to Fig. 2(i1–l1) in the main text. (e–h) Higher-magnification insets of the areas outlined in (a–d). (i–l) Histochemically stained MT regions corresponding to Fig. 2(i2–l2) in the main text. (m–q) Higher-magnification insets of the areas outlined in (i–l).

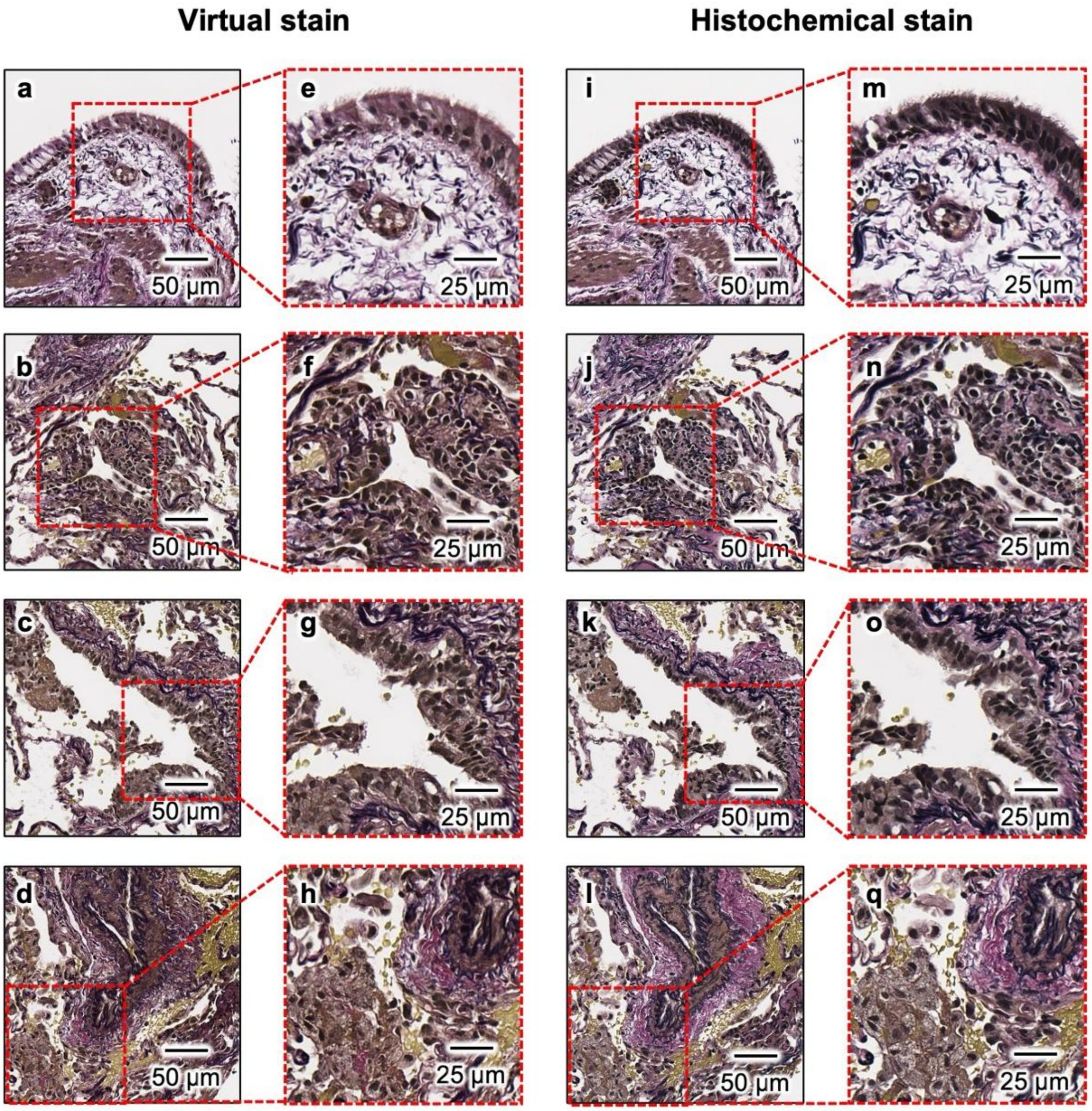

Supplementary Figure 4. Expanded views of the zoomed-in EVG-stained regions from main text Fig. 2. (a–d) Virtually stained EVG regions corresponding to Fig. 2(o1–r1) in the main text. (e–h) Higher-magnification insets of the areas outlined in (a–d). (i–l) Histochemically stained EVG regions corresponding to Fig. 2(o2–r2) in the main text. (m–q) Higher-magnification insets of the areas outlined in (i–l).

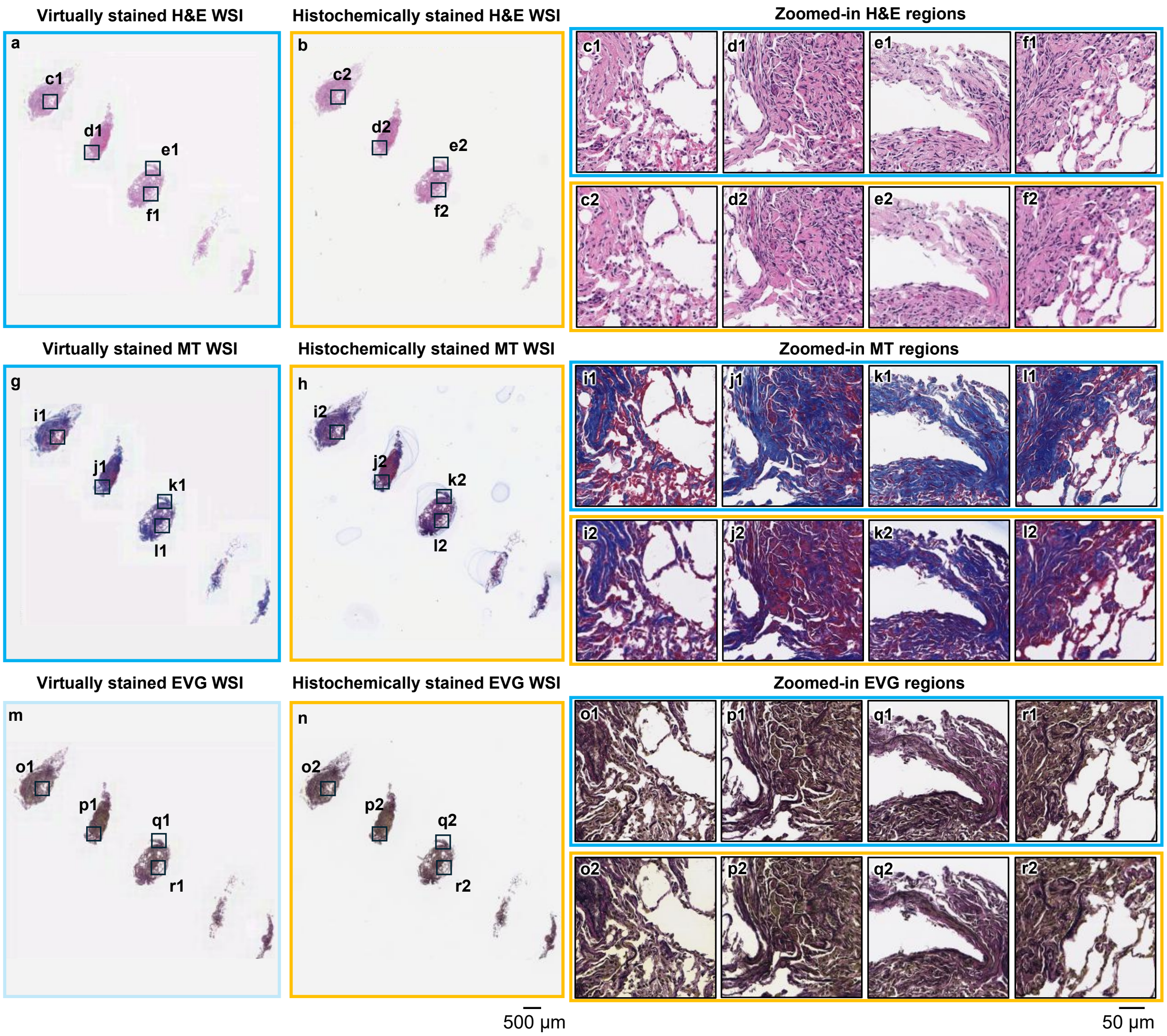

Supplementary Figure 5. Visual comparisons between the virtually stained H&E, MT, and EVG images of a lung transplant patient without rejection and their corresponding histochemically stained counterparts. (a) Virtually stained H&E WSI, which was digitally generated by our virtual staining lung-H&E network by taking label-free autofluorescence images as its input. (b) Histochemical H&E staining results of the same WSI in (a), serving as the ground truth. (c-f) Zoomed-in images of the four exemplary local regions indicated in (a-b), where (c1-f1) are the virtually stained H&E images and (c2-f2) are the corresponding histochemically stained H&E images. (g) Virtually stained MT WSI, which was digitally generated by our virtual staining lung-MT network by taking label-free autofluorescence images as its input. (h) Histochemical MT staining results of the same WSI in (g), serving as the ground truth. (i-l) Zoomed-in images of the four exemplary local regions indicated in (g-h), where (i1-l1) are the virtually stained MT images and (i2-l2) are the corresponding histochemically stained MT images. (m) Virtually stained EVG WSI, which was digitally generated by our virtual staining lung-EVG network by taking label-free autofluorescence images as its input. (n) Histochemical EVG staining results of the same WSI in (m), serving as the ground truth. (o-r) Zoomed-in images of the four exemplary local regions indicated in (m-n), where (o1-r1) are the virtually stained EVG images and (o2-r2) are the corresponding histochemically stained EVG images.

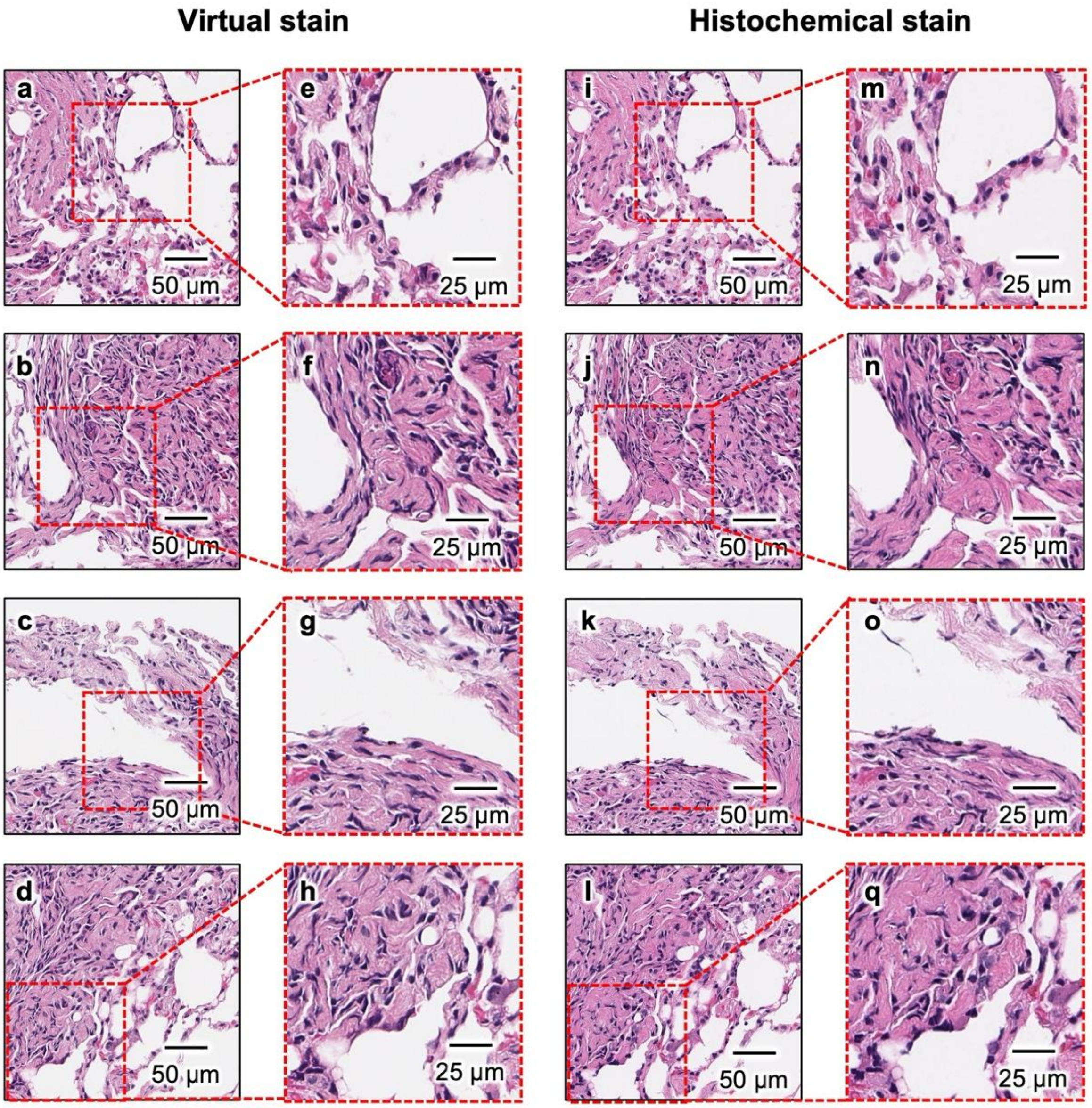

Supplementary Figure 6. Expanded views of the zoomed-in H&E-stained regions from Supplementary Figure 5. (a–d) Virtually stained H&E regions corresponding to Supplementary Figure 5(c1–f1). (e–h) Higher-magnification insets of the areas outlined in (a–d). (i–l) Histochemically stained H&E regions corresponding to Supplementary Figure 5(c2–f2). (m–q) Higher-magnification insets of the areas outlined in (i–l).

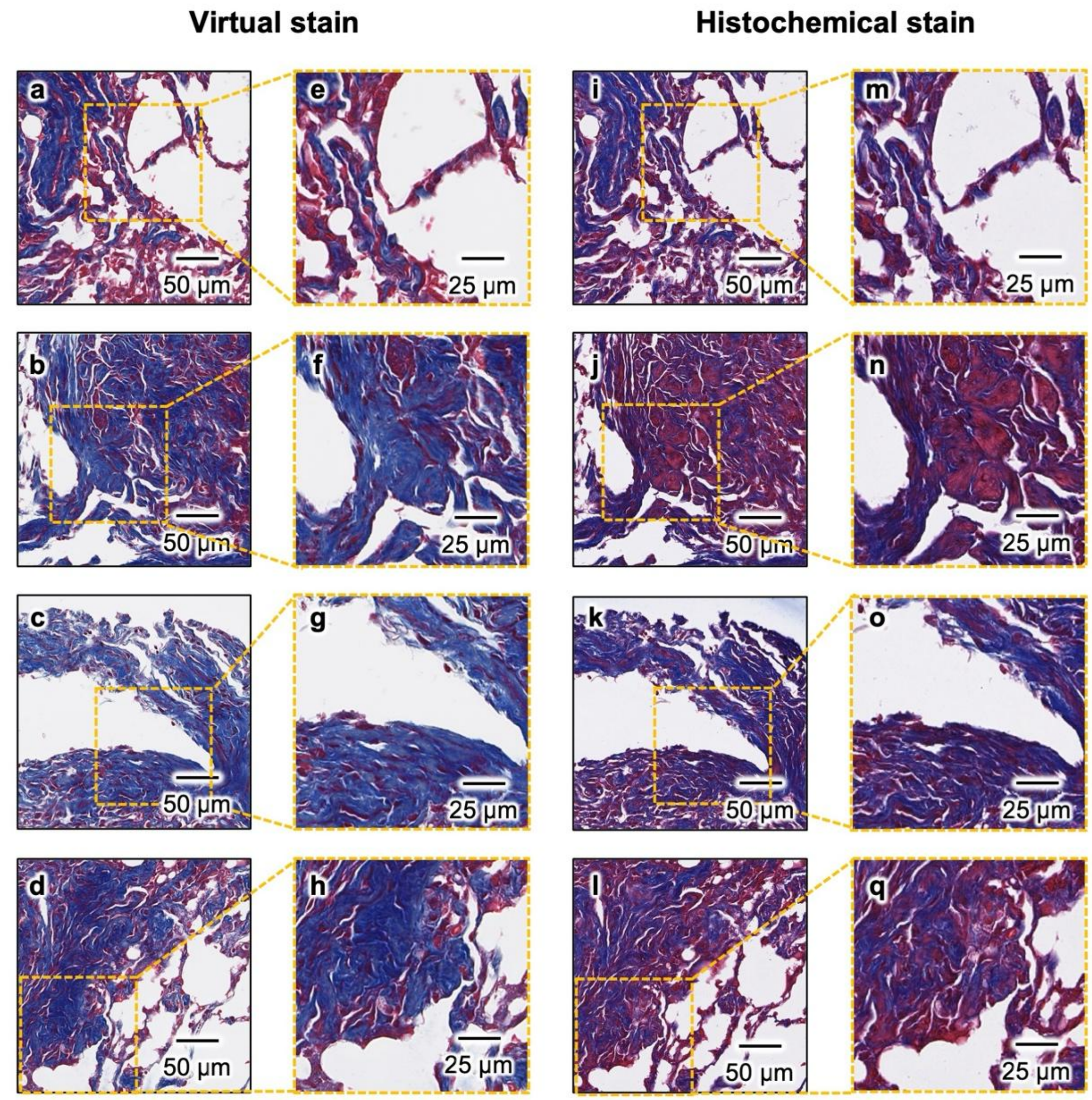

Supplementary Figure 7. Expanded views of the zoomed-in MT-stained regions from Supplementary Figure 5. (a–d) Virtually stained MT regions corresponding to Supplementary Figure 5(i1–l1). (e–h) Higher-magnification insets of the areas outlined in (a–d). (i–l) Histochemically stained MT regions corresponding to Supplementary Figure 5(i2–l2). (m–q) Higher-magnification insets of the areas outlined in (i–l).

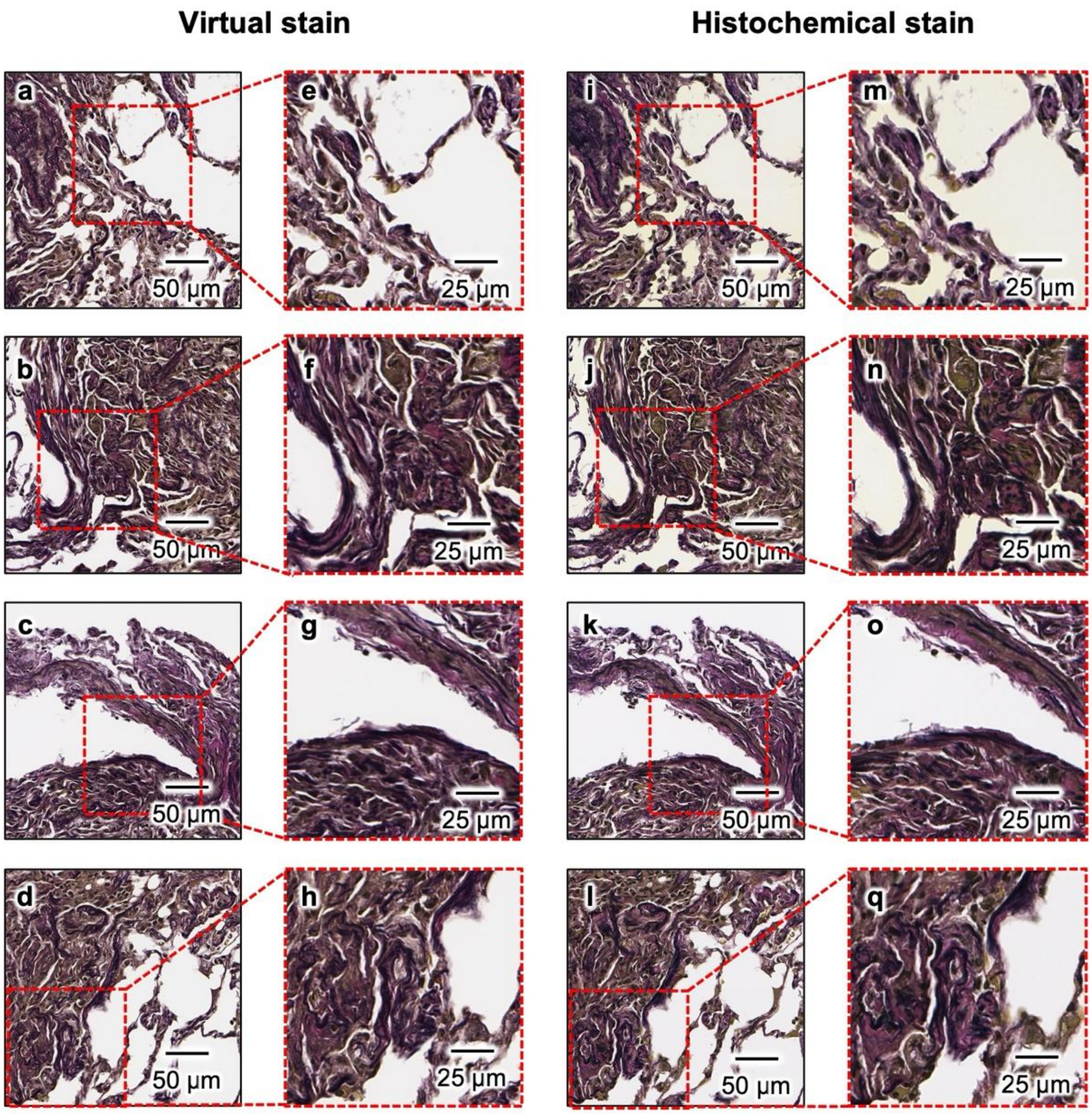

Supplementary Figure 8. Expanded views of the zoomed-in EVG-stained regions from Supplementary Figure 5. (a–d) Virtually stained EVG regions corresponding to Supplementary Figure 5(o1–r1). (e–h) Higher-magnification insets of the areas outlined in (a–d). (i–l) Histochemically stained EVG regions corresponding to Supplementary Figure 5(o2–r2). (m–q) Higher-magnification insets of the areas outlined in (i–l).

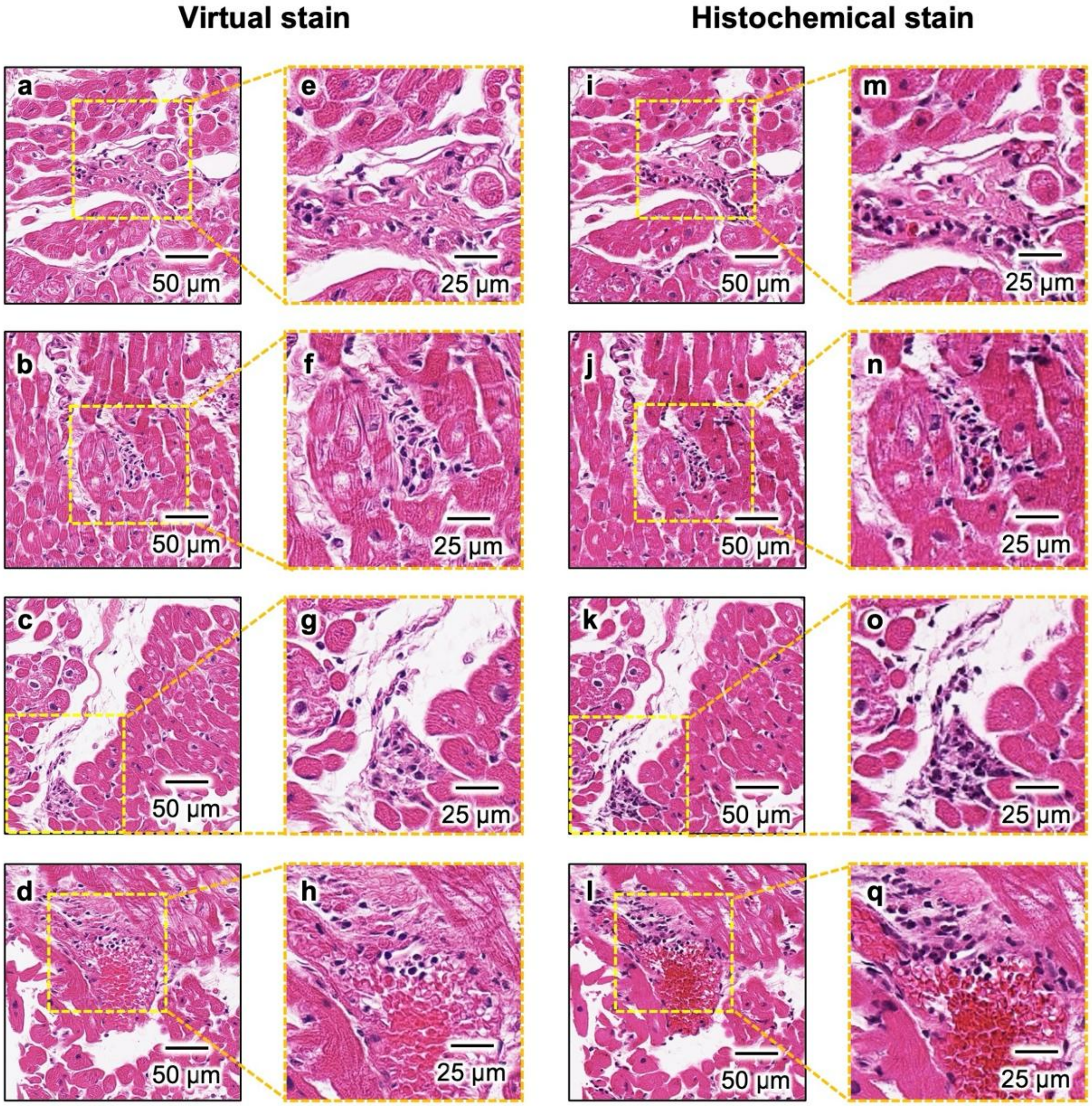

Supplementary Figure 9. Expanded views of the zoomed-in H&E-stained regions from main text Fig. 3. (a–d) Virtually stained H&E regions corresponding to Fig. 3(c1–f1) in the main text. (e–h) Higher-magnification insets of the areas outlined in (a–d). (i–l) Histochemically stained H&E regions corresponding to Fig. 3(c2–f2) in the main text. (m–q) Higher-magnification insets of the areas outlined in (i–l).

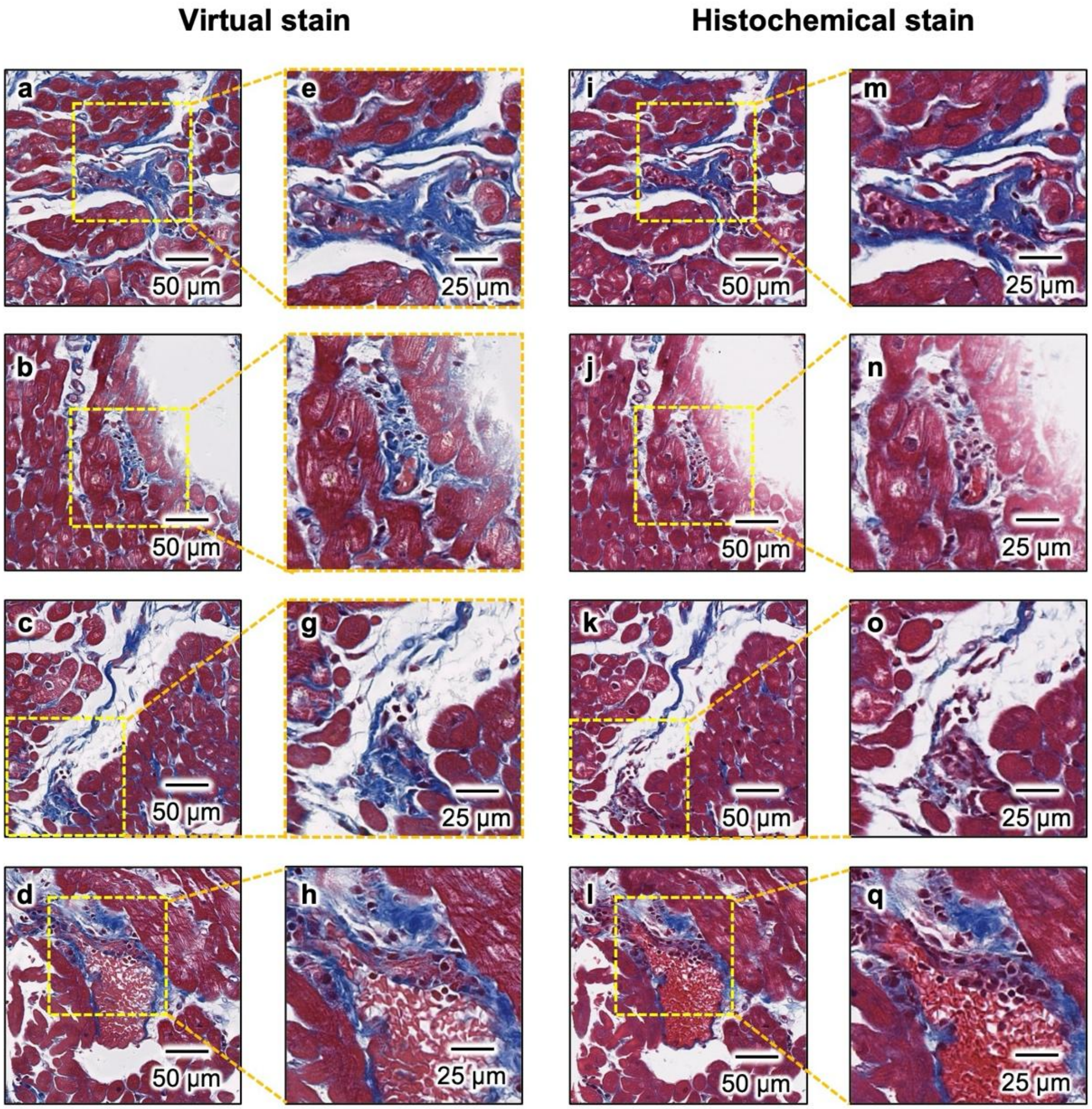

Supplementary Figure 10. Expanded views of the zoomed-in MT-stained regions from main text Fig. 3. (a–d) Virtually stained MT regions corresponding to Fig. 3(i1–l1) in the main text. (e–h) Higher-magnification insets of the areas outlined in (a–d). (i–l) Histochemically stained MT regions corresponding to Fig. 3(i2–l2) in the main text. (m–q) Higher-magnification insets of the areas outlined in (i–l).

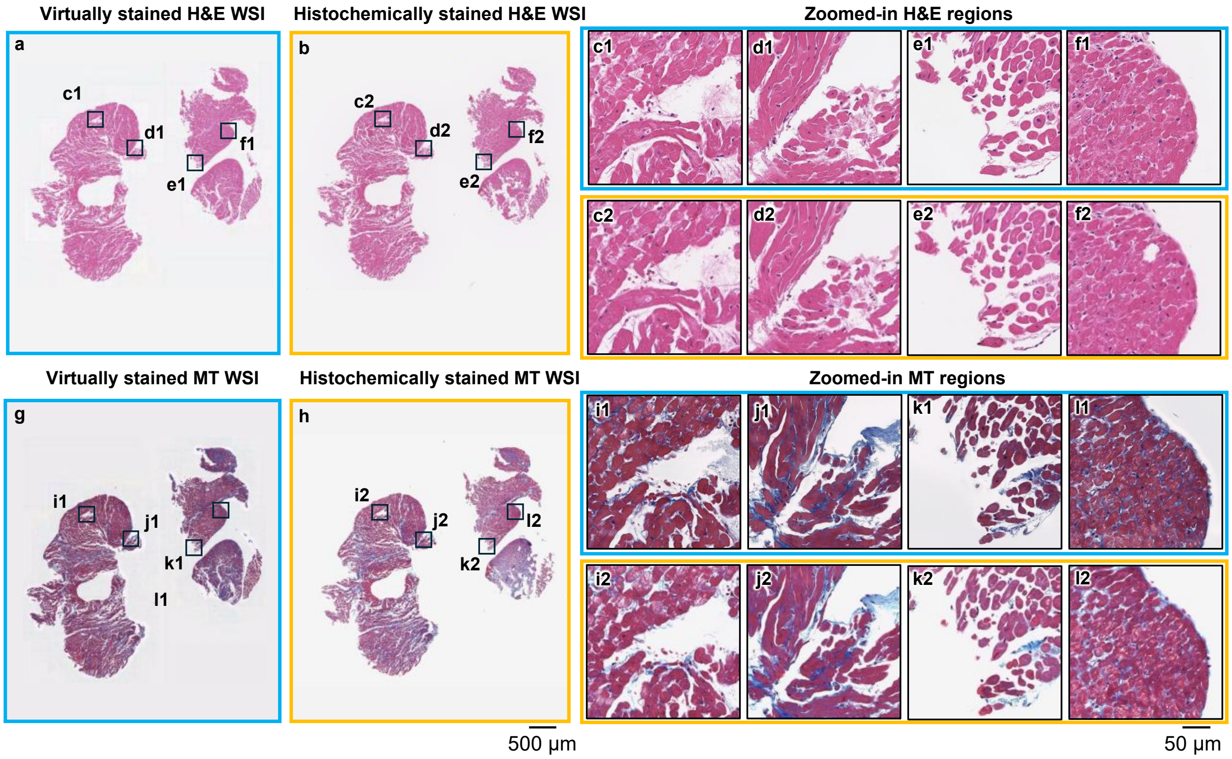

Supplementary Figure 11. Visual comparisons between the virtually stained H&E and MT images of a heart transplant patient without rejection and their corresponding histochemically stained counterparts. (a) Virtually stained H&E WSI, which was digitally generated by our virtual staining heart-H&E network by taking label-free autofluorescence images as its input. (b) Histochemical H&E staining results of the same WSI in (a), serving as the ground truth. (c-f) Zoomed-in images of the four exemplary local regions indicated in (a-b), where (c1-f1) are the virtually stained H&E images and (c2-f2) are the corresponding histochemically stained H&E images. (g) Virtually stained MT WSI, which was digitally generated by our virtual staining heart-MT network by taking label-free autofluorescence images as its input. (h) Histochemical MT staining results of the same WSI in (g), serving as the ground truth. (i-l) Zoomed-in images of the four exemplary local regions indicated in (g-h), where (i1-l1) are the virtually stained MT images and (i2-l2) are the corresponding histochemically stained MT images.

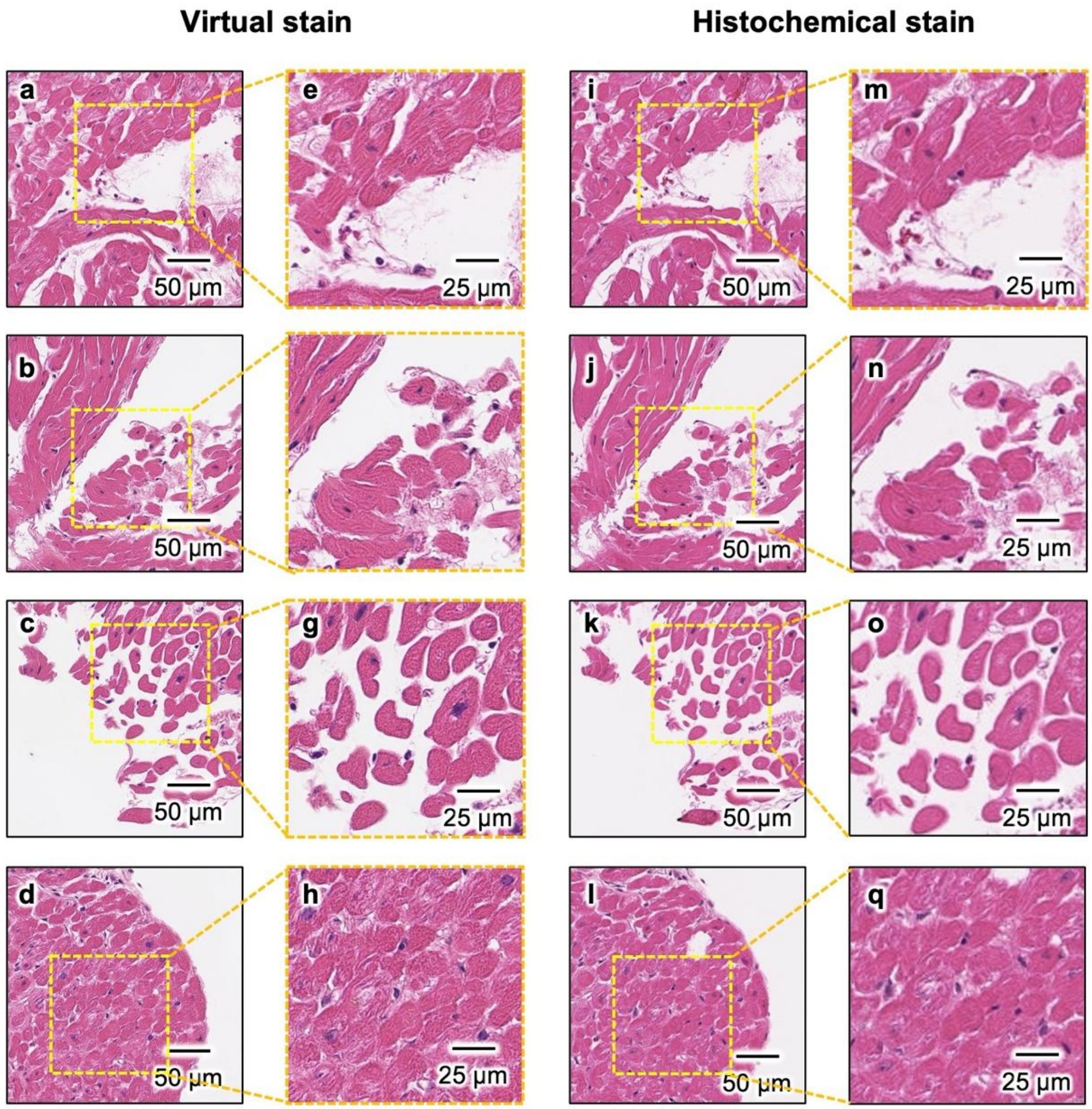

Supplementary Figure 12. Expanded views of the zoomed-in H&E-stained regions from Supplementary Figure 11. (a–d) Virtually stained H&E regions corresponding to Supplementary Figure 11(c1–f1). (e–h) Higher-magnification insets of the areas outlined in (a–d). (i–l) Histochemically stained H&E regions corresponding to Supplementary Figure 11(c2–f2). (m–q) Higher-magnification insets of the areas outlined in (i–l).

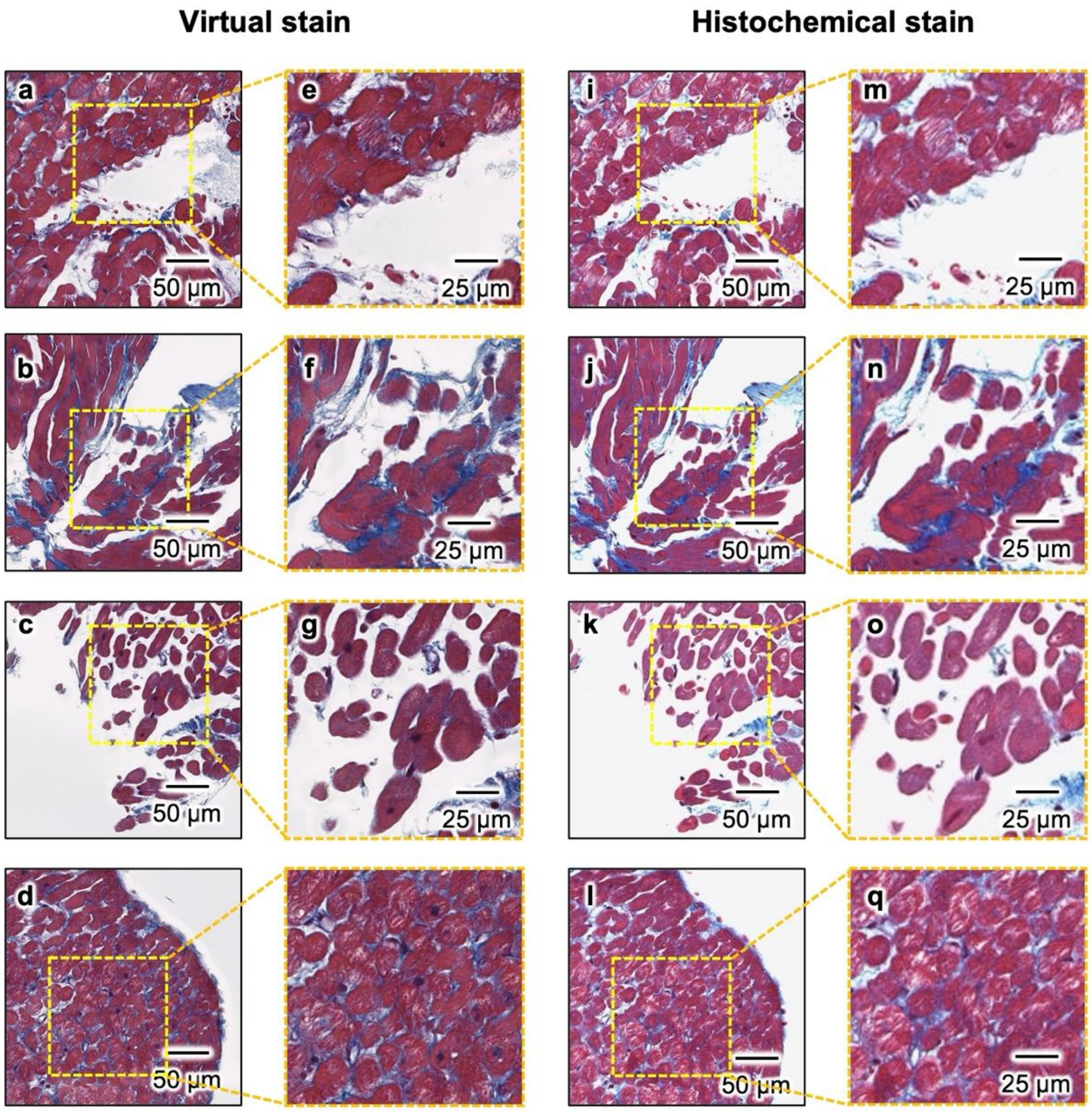

Supplementary Figure 13. Expanded views of the zoomed-in MT-stained regions from Supplementary Figure 11. (a–d) Virtually stained MT regions corresponding to Supplementary Figure 11(i1–l1). (e–h) Higher-magnification insets of the areas outlined in (a–d). (i–l) Histochemically stained MT regions corresponding to Supplementary Figure 11(i2–l2). (m–q) Higher-magnification insets of the areas outlined in (i–l).

VS>HS: The staining quality of VS is significantly better than HS
VS<HS: The staining quality of HS is significantly better than VS
VS≈HS: There is no significant difference between the staining quality of HS and VS

Virtually stained images
Histochemically stained images

a **Staining quality evaluation for lung H&E staining for each pathologist**

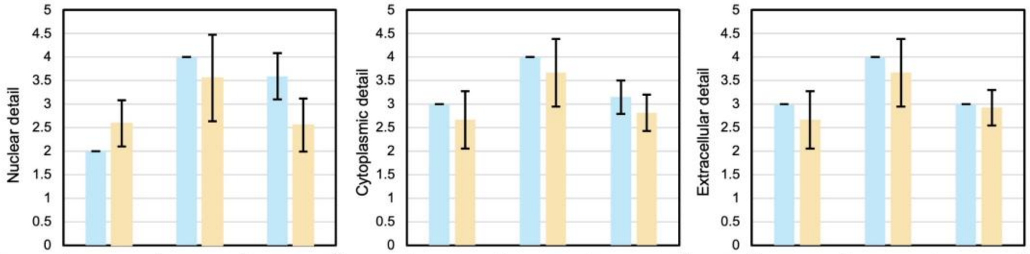

	Pathologist 1	Pathologist 2	Pathologist 3
Mean (VS-HS)	-0.59	0.44	1.04
P value	<0.001	0.026	<0.001
Evaluation	VS<HS	VS>HS	VS>HS

	Pathologist 1	Pathologist 2	Pathologist 3
Mean (VS-HS)	0.33	0.33	0.33
P value	0.010	0.034	0.007
Evaluation	VS>HS	VS>HS	VS>HS

	Pathologist 1	Pathologist 2	Pathologist 3
Mean (VS-HS)	0.33	0.33	0.07
P value	0.010	0.034	0.317
Evaluation	VS>HS	VS>HS	VS≈HS

b **Staining quality evaluation for lung MT staining for each pathologist**

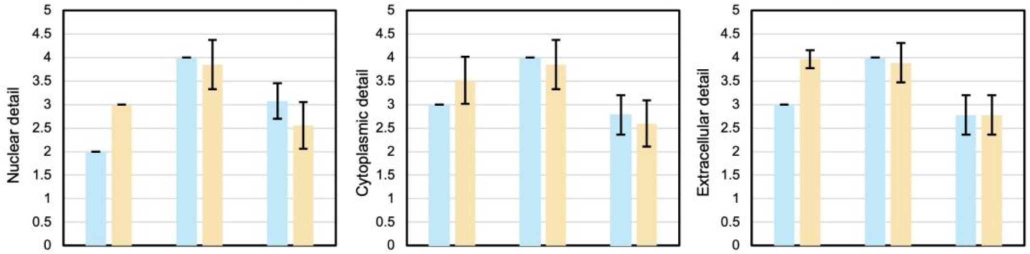

	Pathologist 1	Pathologist 2	Pathologist 3
Mean (VS-HS)	-1.00	0.15	0.52
P value	/	0.157	<0.001
Evaluation	VS<HS	VS≈HS	VS>HS

	Pathologist 1	Pathologist 2	Pathologist 3
Mean (VS-HS)	-0.52	0.15	0.19
P value	<0.001	0.157	0.166
Evaluation	VS<HS	VS≈HS	VS≈HS

	Pathologist 1	Pathologist 2	Pathologist 3
Mean (VS-HS)	-0.96	0.11	0.00
P value	<0.001	0.180	1.000
Evaluation	VS<HS	VS≈HS	VS=HS

c **Staining quality evaluation for lung EVG staining for each pathologist**

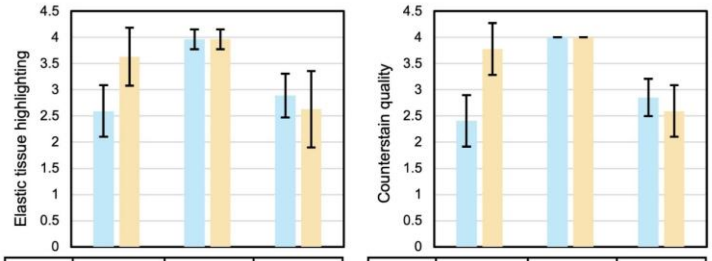

	Pathologist 1	Pathologist 2	Pathologist 3
Mean (VS-HS)	-1.04	0.00	0.26
P value	<0.001	1.000	0.100
Evaluation	VS<HS	VS=HS	VS≈HS

	Pathologist 1	Pathologist 2	Pathologist 3
Mean (VS-HS)	-1.37	0.00	0.26
P value	<0.001	/	0.035
Evaluation	VS<HS	VS=HS	VS>HS

Supplementary Figure 14. Score-based quantitative per-pathologist evaluations for assessing the virtual staining results and their corresponding histochemical ground truth images for lung transplant patients, together with the statistical analysis. (a) Staining quality scores of virtually and histochemically stained H&E WSIs evaluated by each of the three board-certified pathologists, along with the statistical evaluation results of the two-tailed, paired Wilcoxon signed-rank test. The mean and standard deviation values of these scores for each metric and pathologist were calculated across all the histochemically stained high-quality lung test WSIs (n=27). (b) same as in (a), except for the MT stain. (c) same as in (a) and (b), except for the EVG stain.

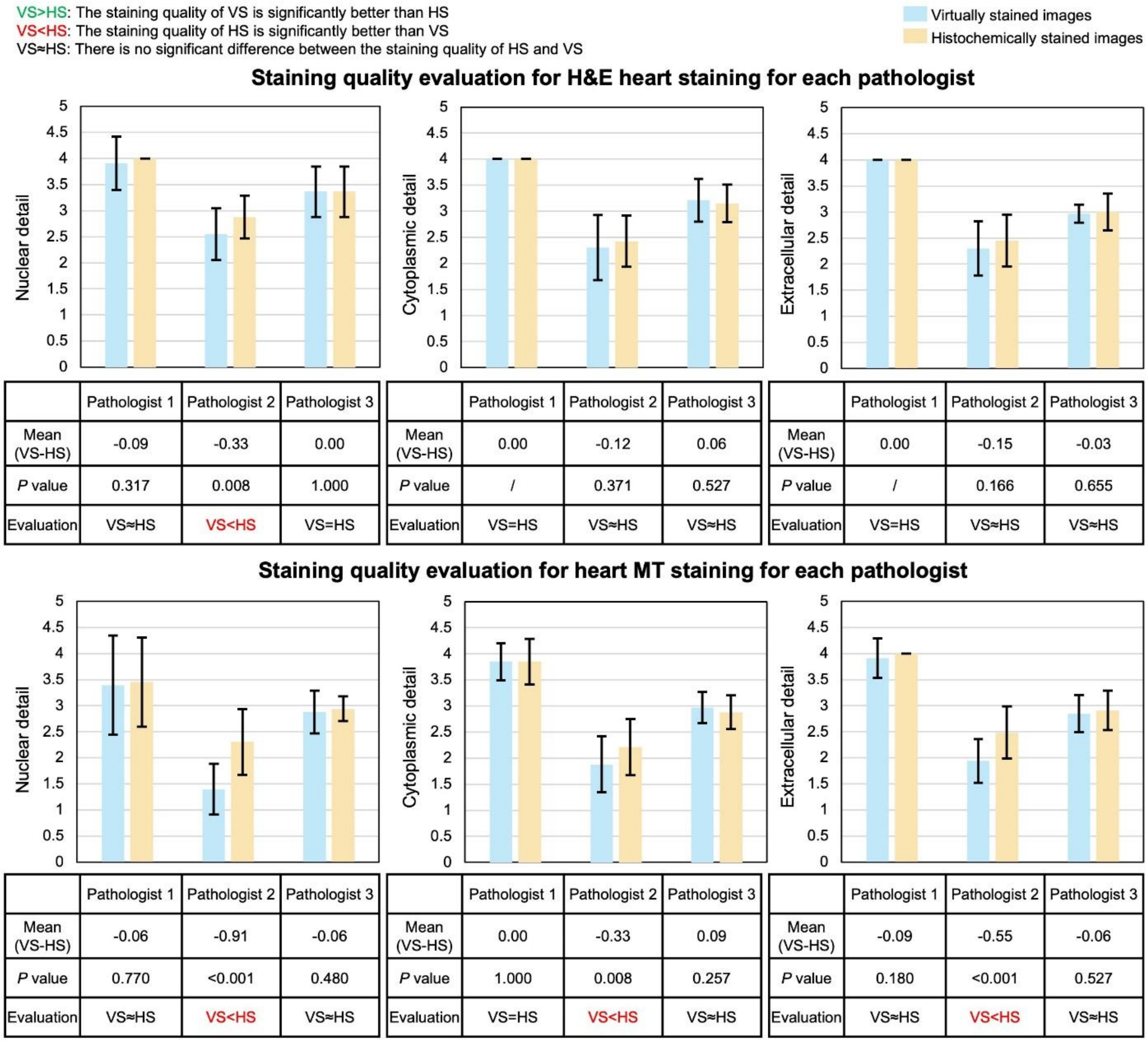

H&E – Nuclear detail

	Pathologist 1	Pathologist 2	Pathologist 3
Mean (VS-HS)	-0.09	-0.33	0.00
P value	0.317	0.008	1.000
Evaluation	VS≈HS	VS<HS	VS=HS

H&E – Cytoplasmic detail

	Pathologist 1	Pathologist 2	Pathologist 3
Mean (VS-HS)	0.00	-0.12	0.06
P value	/	0.371	0.527
Evaluation	VS=HS	VS≈HS	VS≈HS

H&E – Extracellular detail

	Pathologist 1	Pathologist 2	Pathologist 3
Mean (VS-HS)	0.00	-0.15	-0.03
P value	/	0.166	0.655
Evaluation	VS=HS	VS≈HS	VS≈HS

MT – Nuclear detail

	Pathologist 1	Pathologist 2	Pathologist 3
Mean (VS-HS)	-0.06	-0.91	-0.06
P value	0.770	<0.001	0.480
Evaluation	VS≈HS	VS<HS	VS≈HS

MT – Cytoplasmic detail

	Pathologist 1	Pathologist 2	Pathologist 3
Mean (VS-HS)	0.00	-0.33	0.09
P value	1.000	0.008	0.257
Evaluation	VS=HS	VS<HS	VS≈HS

MT – Extracellular detail

	Pathologist 1	Pathologist 2	Pathologist 3
Mean (VS-HS)	-0.09	-0.55	-0.06
P value	0.180	<0.001	0.527
Evaluation	VS≈HS	VS<HS	VS≈HS

Supplementary Figure 15. Score-based quantitative per-pathologist evaluations for assessing the virtual staining results and their corresponding histochemical ground truth images for heart transplant patients, together with the statistical analysis. (a) Staining quality scores of virtually and histochemically stained H&E WSIs evaluated by each of the three board-certified pathologists, along with the statistical evaluation results of the two-tailed, paired Wilcoxon signed-rank test. The mean and standard deviation values of these scores for each metric and pathologist were calculated across all the histochemically stained high-quality heart test WSIs (n=33). (b) same as in (a), except for the MT stain.

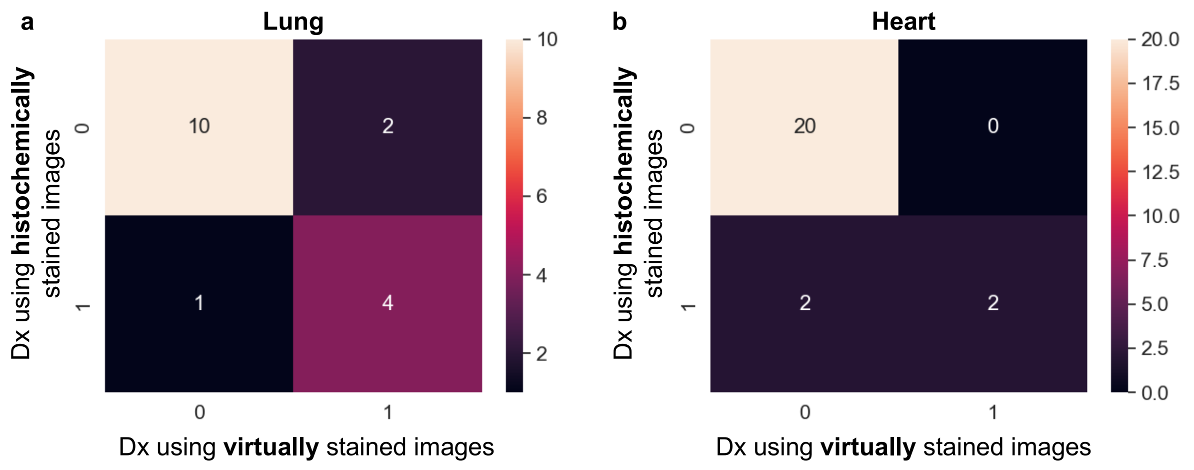

Supplementary Figure 16. Confusion matrices of diagnostic results based on the virtually stained WSIs vs. diagnostic results based on their corresponding histochemically stained WSIs for (a) lung, (b) heart transplant biopsies.

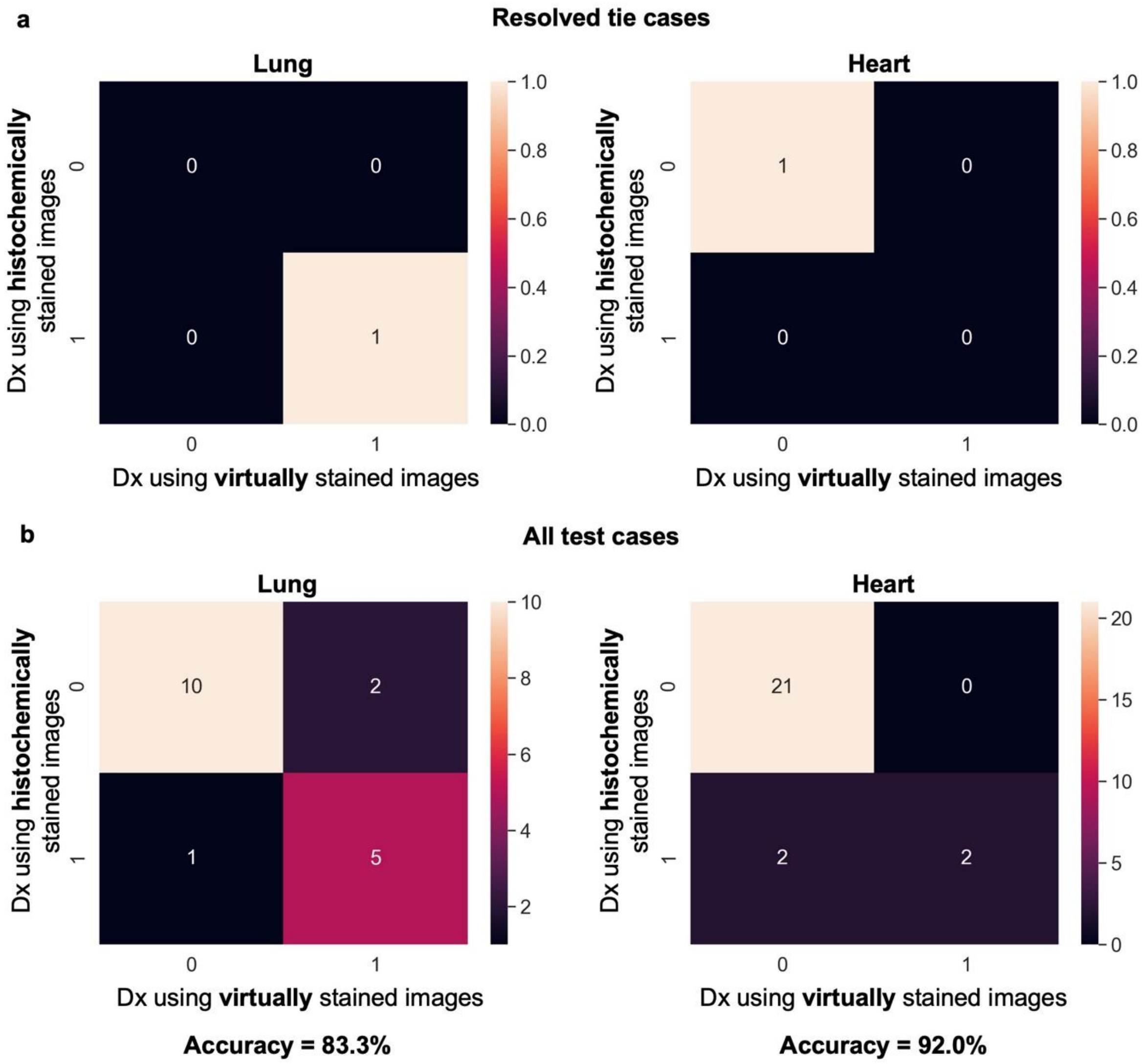

Supplementary Figure 17. Confusion matrices comparing diagnoses from virtually stained vs. histochemically stained WSIs when considering tie cases (2-2 votes). (a) Resolved tie case only confusion matrices for lung (left) and heart (right), where 2 tie cases were resolved by senior pathologist consensus (i.e., 2 senior pathologists of the group of 4 pathologists agreed on these cases). (b) Confusion matrices for the full test sets after incorporating those resolved tie cases, yielding overall accuracies of 83.3% for lung and 92.0% for heart.

N: non-rejection (negative)
Y: rejection (positive)
N/A: not applicable

VS/HS_Px: The diagnostic outcomes provided by the x-th pathologist using virtually stained (VS) or histochemically stained (HS) WSIs
VS/HS_Final: Final diagnostic outcomes after decisive voting (3-0 or 3-1 votes) using virtually stained (VS) or histochemically stained (HS) WSIs

Case #	VS_P1	VS_P2	VS_P3	VS_P4	VS_Final	HS_P1	HS_P2	HS_P3	HS_P4	HS_Final
1	N	N	Y	N	N	N	N	N	N/A	N
2	N	N	Y	N	N	N	N	N	N/A	N
3	N	Y	Y	N	N/A	N	N	Y	N	N
4	N	N	Y	N	N	N	N	N	N/A	N
5	N	N	N	N/A	N	N	N	Y	N	N
6	N	N	Y	N	N	N	N	N	N/A	N
7	N	N	Y	N	N	N	N	N	N/A	N
8	N	N	N	N/A	N	N	N	N	N/A	N
9	N	Y	Y	Y	Y	N	N	Y	N	N
10	N	N	N	N/A	N	N	N	N	N/A	N
11	N	Y	Y	Y	Y	N	Y	Y	Y	Y
12	Y	Y	Y	N/A	Y	Y	Y	Y	N/A	Y
13	N	Y	Y	Y	Y	Y	Y	Y	N/A	Y
14	N	N	Y	N	N	N	Y	N	N	N
15	N	N	Y	N	N	N	N	N	N/A	N
16	N	N	Y	N	N	N	Y	Y	Y	Y
17	N	N	Y	Y	N/A	N	N	N	N/A	N
18	N	N	Y	Y	N/A	N	N	Y	N	N
19	Y	Y	Y	N/A	Y	Y	Y	Y	N/A	Y
20	N	N	Y	Y	N/A	N	N	N	N/A	N
21	N	Y	Y	Y	Y	N	Y	N	Y	N/A
22	N	Y	Y	Y	Y	N	Y	N	N	N
23	N	Y	Y	N	N/A	N	N	Y	N	N

Supplementary Figure 18. Case-by-case diagnostic results from every pathologist using the virtually stained WSIs and the histochemically stained WSIs of lung transplant patients. The yellow-shaded cases (17 cases in total) represent those with decisive voting preferences (3-0 or 3-1 votes), while the non-yellow-shaded cases, which had an even split (2-2 votes) for diagnoses using either virtually stained WSIs or histochemically stained WSIs, were excluded from the final diagnostic results since a comparative decision could not be made.

N: non-rejection (negative)
Y: rejection (positive)
N/A: not applicable

VS/HS_Px: The diagnostic outcomes provided by the x-th pathologist using virtually stained (VS) or histochemically stained (HS) WSIs
VS/HS_Final: Final diagnostic outcomes after decisive voting (3-0 or 3-1 votes) using virtually stained (VS) or histochemically stained (HS) WSIs

Case #	VS_P1	VS_P2	VS_P3	VS_P4	VS_Final	HS_P1	HS_P2	HS_P3	HS_P4	HS_Final
1	N	N	N	N/A	N	Y	N	N	N	N
2	N	N	N	N/A	N	Y	N	N	Y	N/A
3	N	N	N	N/A	N	N	N	N	N/A	N
4	N	N	N	N/A	N	N	N	N	N/A	N
5	N	N	N	N/A	N	Y	N	N	N	N
6	Y	Y	Y	N/A	Y	Y	Y	Y	N/A	Y
7	Y	N	N	N	N	Y	N	N	N	N
8	N	N	Y	N	N	N	Y	Y	N	N/A
9	Y	Y	Y	N/A	Y	Y	Y	Y	N/A	Y
10	N	N	N	N/A	N	N	N	N	N/A	N
11	N	N	N	N/A	N	Y	N	N	Y	N/A
12	N	N	N	N/A	N	N	Y	Y	Y	Y
13	N	N	N	N/A	N	N	N	N	N/A	N
14	N	N	N	N/A	N	N	N	N	N/A	N
15	N	N	N	N/A	N	N	N	N	N/A	N
16	N	N	N	N/A	N	N	N	N	N/A	N
17	N	N	N	N/A	N	N	N	N	N/A	N
18	Y	N	Y	N	N/A	N	N	N	N/A	N
19	N	N	N	N/A	N	N	N	N	N/A	N
20	N	N	Y	N	N	Y	Y	Y	N/A	Y
21	N	N	N	N/A	N	N	N	N	N/A	N
22	N	N	N	N/A	N	N	N	N	N/A	N
23	Y	N	N	N	N	N	N	N	N/A	N
24	N	N	N	N/A	N	N	N	N	N/A	N
25	N	N	N	N/A	N	N	N	N	N/A	N
26	N	N	Y	N	N	Y	N	N	N	N
27	N	N	N	N/A	N	N	N	N	N/A	N
28	N	N	N	N/A	N	N	N	N	N/A	N

Supplementary Figure 19. Case-by-case diagnostic results from every pathologist using the virtually stained WSIs and the histochemically stained WSIs of heart transplant patients. The yellow-shaded cases (24 cases in total) represent those with decisive voting preferences (3-0 or 3-1 votes), while the non-yellow-shaded cases, which had an even split (2-2 votes) for diagnoses using either virtually stained WSIs or histochemically stained WSIs, were excluded from the final diagnostic results since a comparative decision could not be made.

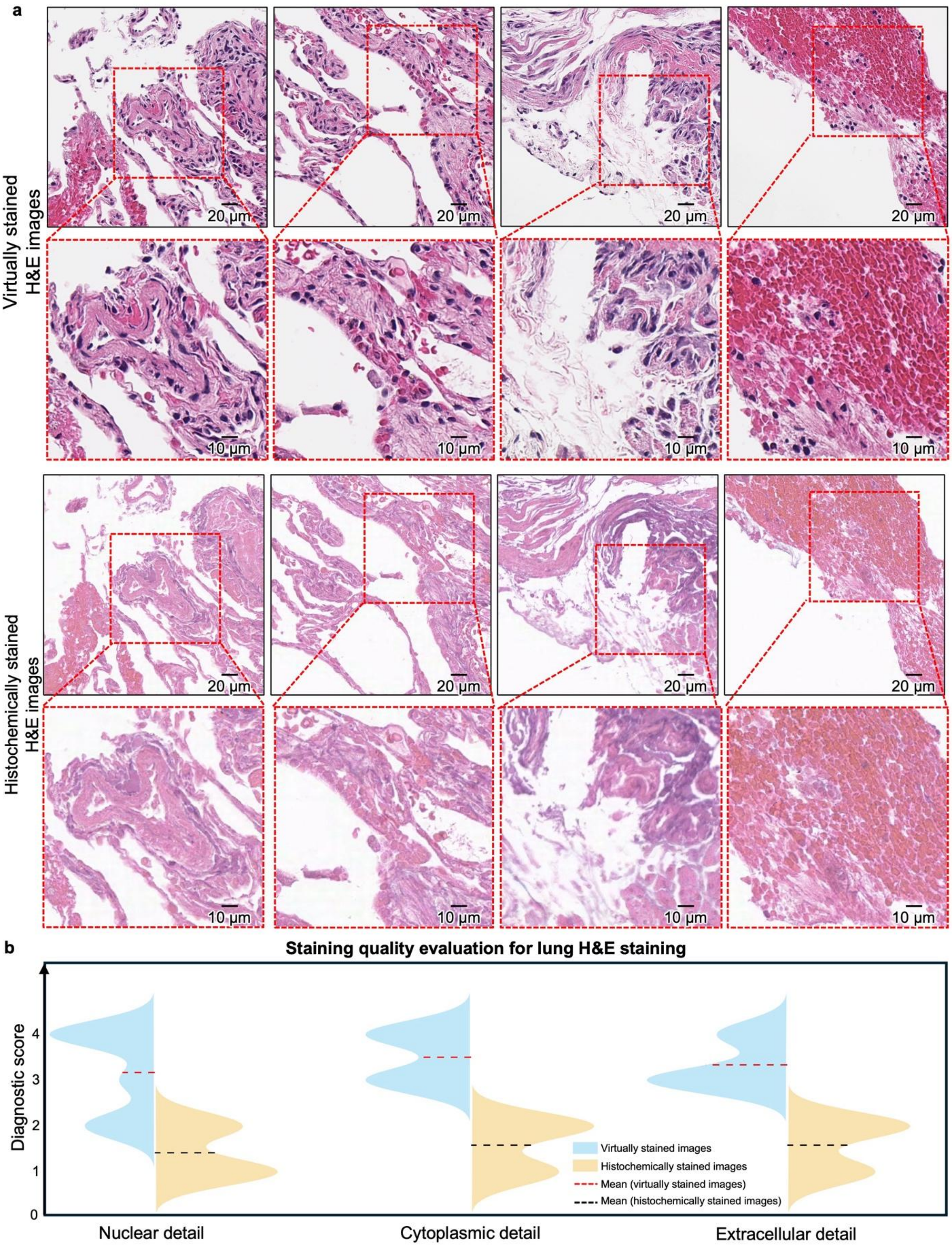

Supplementary Figure 20. Comparisons between the virtually stained H&E images of lung transplant patients and their corresponding histochemically stained counterparts, which suffered from prominent chemical staining artifacts. (a) Top:

Virtually stained H&E images, digitally generated by our virtual staining lung-H&E network by taking label-free autofluorescence images as its input. Bottom: Histochemical H&E staining results of the same FOVs shown on the top, which have extensive pale, under-stained areas and lack well-defined nuclear contours. (b) Violin plots showing the staining quality scores of virtually and histochemically stained lung H&E images evaluated by three board-certified pathologists from the aspects of nuclear detail, cytoplasmic detail, and extracellular detail. The mean values of these scores for each metric were calculated across four lung cases with prominent histochemical staining failures (not seen in the training) and all three pathologists ($n=4\times3=12$).

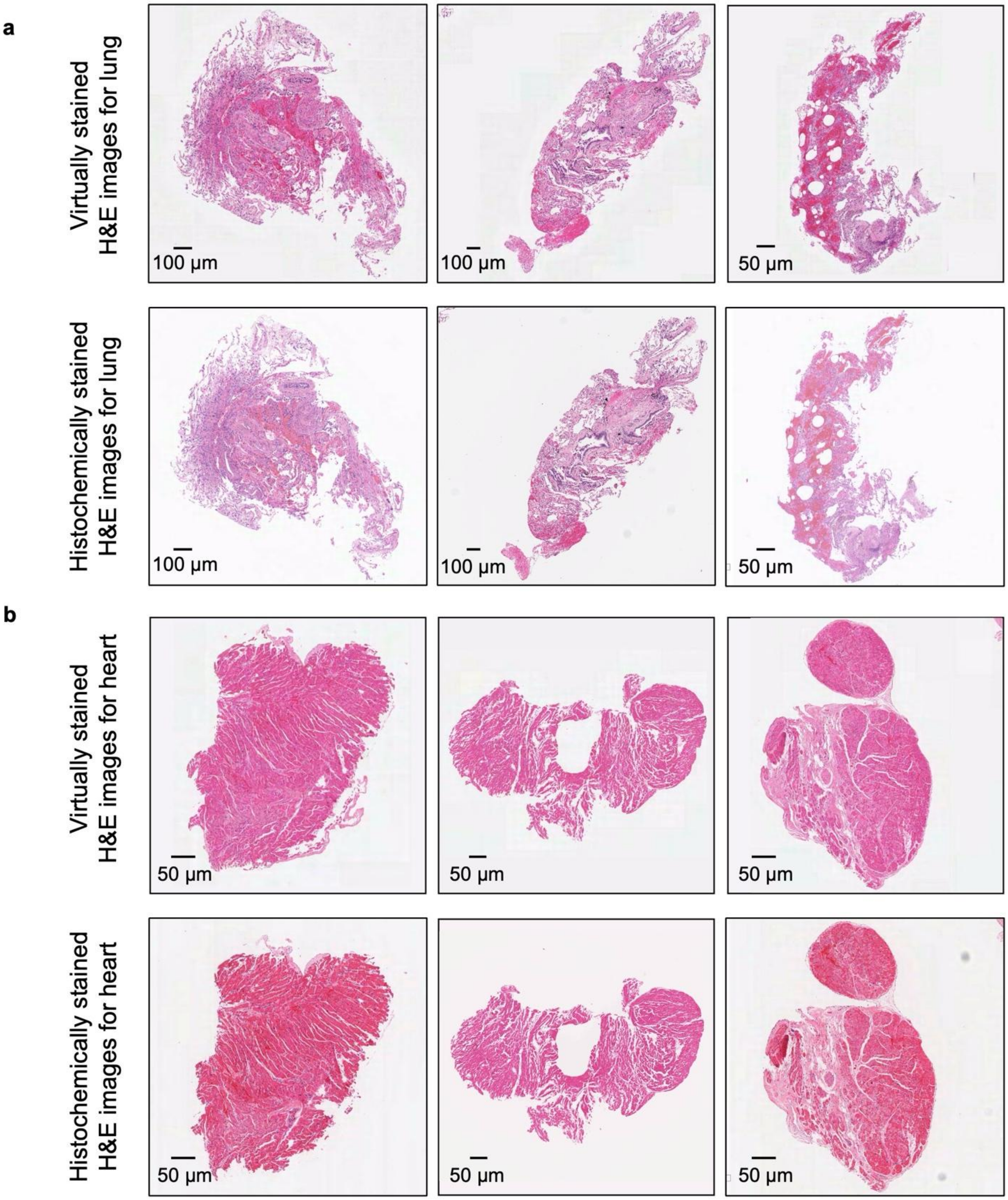

Supplementary Figure 21. Comparisons between the virtually stained H&E images with a uniform and standardized color style and their corresponding histochemically stained counterparts with a large stain variability for (a) lung, (b) heart.

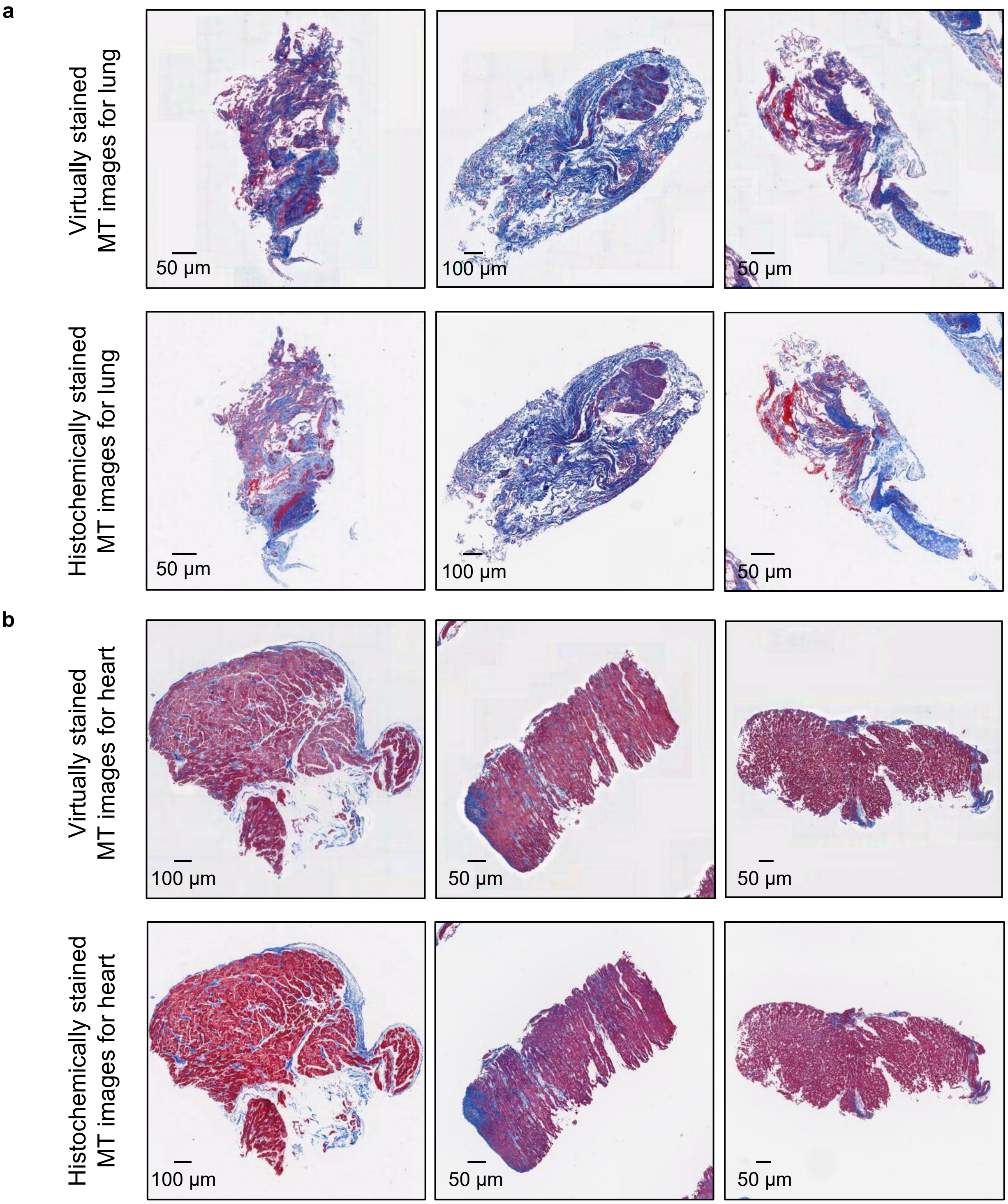

Supplementary Figure 22. Comparisons between the virtually stained MT images with a uniform and standardized color style and their corresponding histochemically stained counterparts with a large stain variability for (a) lung, (b) heart.

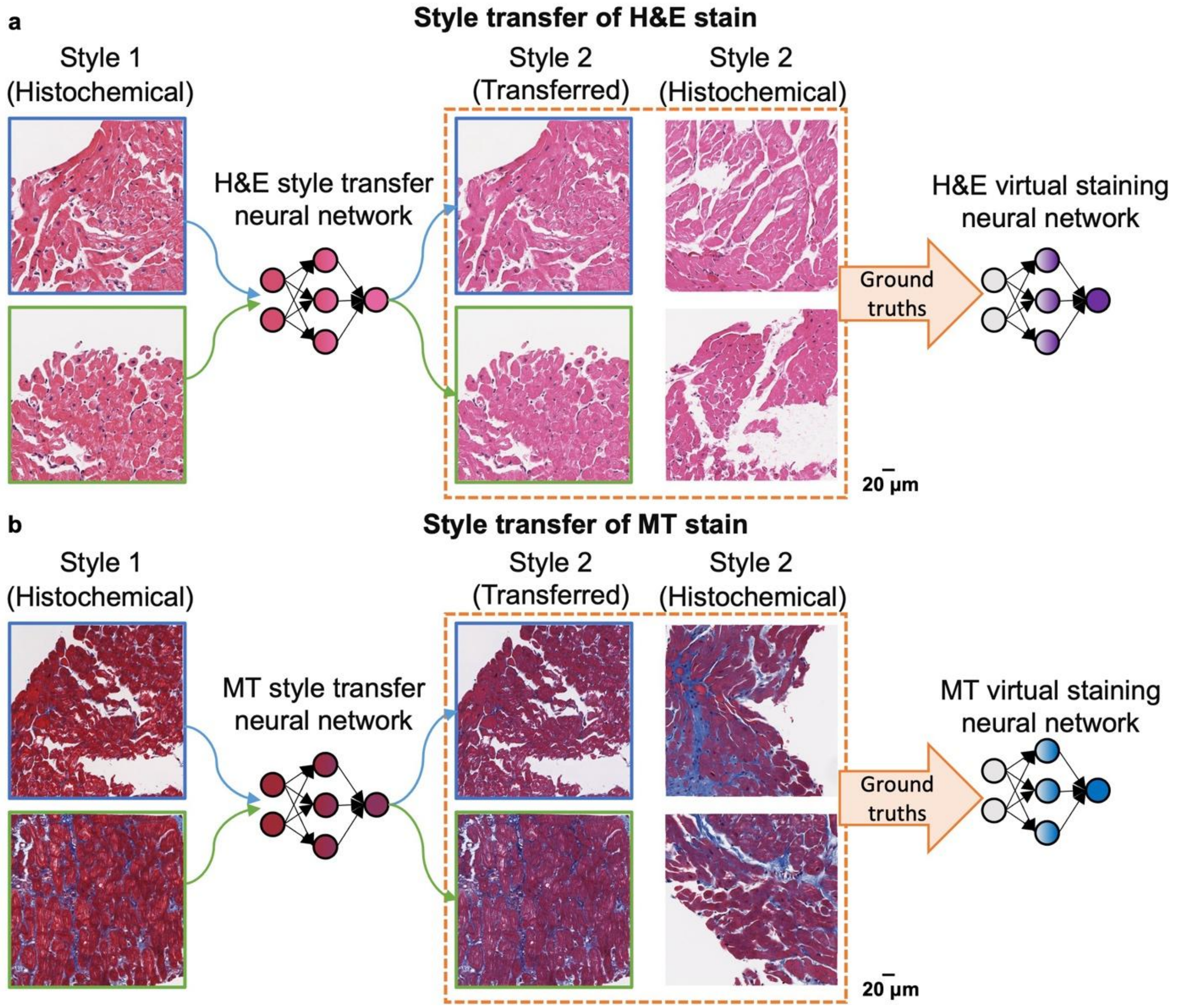

Supplementary Figure 23. Style transfer of the histochemically stained images from the first institution (Cedars-Sinai Medical Center) to match the color style of the second institution (UCLA TPCL) for (a) H&E stain and (b) MT stain. These transformed images, along with the original histochemically stained images from the second institution, served as ground truth images for training the virtual staining networks.
